# Improved Battery Cycle Life Prediction Using a Hybrid Data‐Driven Model Incorporating Linear Support Vector Regression and Gaussian

**DOI:** 10.1002/cphc.202100829

**Published:** 2022-03-01

**Authors:** Mohammad Alipour, Shiva Sander Tavallaey, Anna M. Andersson, Daniel Brandell

**Affiliations:** ^1^ Department of Chemistry – Ångström Laboratory Uppsala University 751 21 Uppsala Sweden; ^2^ ABB AB Corporate Research Forskargränd 7 SE-721 78 Västerås Sweden; ^3^ Department of Mechanics School of Science KTH SE-100 44 Stockholm Sweden

**Keywords:** battery cycle life, cycle life prediction, data-driven modeling, linear support vector regression, Gaussian process regression

## Abstract

The ability to accurately predict lithium‐ion battery life‐time already at an early stage of battery usage is critical for ensuring safe operation, accelerating technology development, and enabling battery second‐life applications. Many models are unable to effectively predict battery life‐time at early cycles due to the complex and nonlinear degrading behavior of lithium‐ion batteries. In this study, two hybrid data‐driven models, incorporating a traditional linear support vector regression (LSVR) and a Gaussian process regression (GPR), were developed to estimate battery life‐time at an early stage, before more severe capacity fading, utilizing a data set of 124 battery cells with lifetimes ranging from 150 to 2300 cycles. Two type of hybrid models, here denoted as A and B, were proposed. For each of the models, we achieved 1.1 % (A) and 1.4 % (B) training error, and similarly, 8.3 % (A) and 8.2 % (B) test error. The two key advantages are that the error percentage is kept below 10 % and that very low error values for the training and test sets were observed when utilizing data from only the first 100 cycles.The proposed method thus appears highly promising for predicting battery life during early cycles.

## Graphical abstract

**A novel hybrid data‐driven model** combining linear support vector regression (LSVR) and Gaussian process regression (GPR) is proposed for estimating battery life‐time at an early stage. The LSVR model is used to estimate battery cycle life, whereas the GPR model is used to predict the cycle life residual, which is the difference between the real and the LSVR model‘s predicted cycle life. The final prediction is the sum of the LSVR′s predicted cycle life and the GPR′s predicted cycle‐life residual.

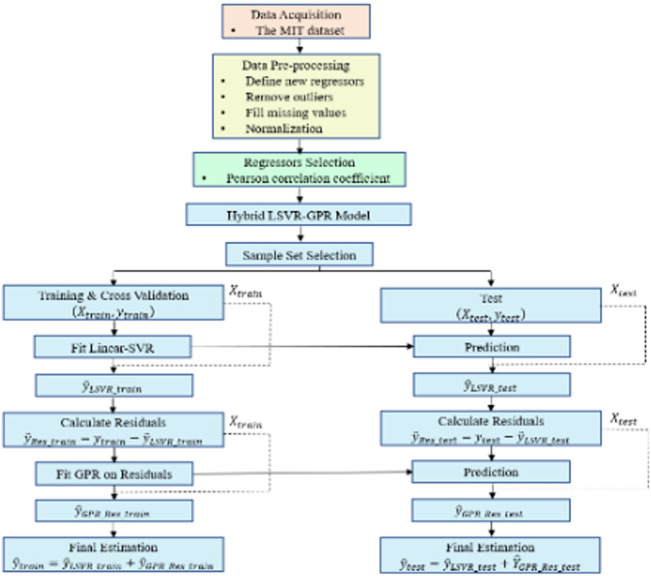

## 1 Introduction

Lithium‐ion (Li‐ion) batteries are used in a wide range of applications, from electronic devices to electric vehicles and grid energy storage systems, because of their low cost, long life, and high energy density.[^1^, ^2^] These rechargeable batteries lose capacity, energy, and power over time as a result of internal electrochemical processes and external operating conditions. Thus, Li‐ion battery aging is generally characterized as an increase in internal resistance and a decrease in capacity, which constitute major problems.[^3^, ^4^] Battery aging increases the cost of energy storage systems and may potentially result in serious accidents such as fires and explosions. Therefore, accurate battery cycle life prediction is critical for optimizing the performance of energy storage systems while assuring their safety and reliability.^[5]^

Since the emergence of the commercial electric vehicles (EVs), battery life‐time has been a focus of research, with different Li‐ion batteries being cycled and/or stored in order to identify different degradation mechanisms.^[6]^ To maintain the safety and reliability of battery‐powered systems, it is generally recommended that batteries should be replaced when they can only store 80 % of their initial capacity. Laboratory studies are typically performed to better understand battery aging behavior under various operating conditions, with the resulting data being fed into or used to develop battery cycle life prediction models.^[7]^ In recent years, a variety of methods for predicting battery lifetime have been presented.[^8^, ^9^, ^10^] Generally, battery life‐time prediction methods include model‐based, data‐driven, and hybrid approaches.[^11^, ^12^, ^13^, ^14^] Model‐based approaches use information of a system's failure mechanisms (e. g., solid electrolyte interface (SEI) growth) to provide a mathematical description of the degradation process, or they build an empirical model (experience‐based models) to reproduce the system's declining trajectory.^[15]^ They normally use different filtering algorithms such as the Kalman filter (KF),^[16]^ the extended Kalman filter (EKF),^[17]^ or the particle filter (PF)^[18]^ to update model parameters recursively by sampling one measurement data point at a time. Hu et al.,^[19]^ for example, used a dual fractional‐order extended Kalman filter (DFOEKF) for co‐estimation of state of charge (SOC) and state of health (SOH) for Lithium‐ion batteries. Data‐driven modeling strategies, on the other hand, use historical data, real‐time data, or both to determine the characteristics of the currently observed damage state and estimate future trends.[^12^, ^20^, ^21^, ^22^] Ng et al.^[23]^ published a list of the recent data‐driven models for battery state estimation. Finally, hybrid approaches combine model‐based and data‐driven methods in order to leverage the strengths of both approaches.[^11^, ^15^, ^24^, ^25^]

Data‐driven models using statistical and machine learning techniques have gained a lot of interest in battery prognostic applications since they do not necessitate a deep understanding of battery failure and other physical mechanisms. In these models, the battery systems are treated as black box systems to provide a mapping between various input and output variables. An increasing number of articles has been devoted to data‐driven algorithms for predicting battery state and life‐time in recent years. Che et al.^[26]^ used a universal deep learning method for prognostic and battery pack state of health estimation. Hu et al.^[27]^ developed a hybrid approach for lithium‐ion battery RUL prediction based on particle filter (PF) and long short‐term memory (LSTM) neural network. Liu et al.^[28]^ employed a Gaussian process regression (GPR) with composite kernels coupling the Arrhenius law and a polynomial equation to capture the electrochemical and empirical knowledge of battery degradation. Nuhic et al.^[29]^ used the support vector machine (SVM) for the estimation of state of health (SOH) and the remaining useful life (RUL). Ma et al.^[30]^ used the battery capacity in a specific window (the minimum embedding dimensions of the capacity data) as input features, and created a hybrid neural network that integrated a convolutional neural network and long short‐term memory to predict battery life‐time. Son et al.^[31]^ employed a Gaussian process regression using multiphysics features including mechanical and impedance evolutionary responses to estimate the SOH of batteries. Even though these present methods provide satisfactory results in terms of battery life‐time prediction, they often require data corresponding to at least 25 % aging in order to accurately estimate the target value. Due to the non‐linear and complex degradation process of Li‐ion batteries, precisely estimating battery life‐time at early cycles – where the battery is largely yet to exhibit capacity degradation ‐ is more challenging.

This paper offers two hybrid models combining a linear support vector regression (LSVR) and a Gaussian process regression (GPR) for battery cycle‐life prediction using data from only the first 100 cycles in a data set^[32]^ of 124 cells with lifetimes ranging from 150 to 2300 cycles. The paper is organized as follows: In section 2, a comprehensive mathematical description of the proposed hybrid data‐driven model is given. In section 3, the methodologies including the data description, the data preprocessing, the model development, and the model assessment methods are reviewed. Section 4 shows the results of the battery cycle‐life prediction and compares them to published data.^[32]^ The paper is concluded in section 5.

## 2 Theory

### 2.1 Regression

Supervised learning can be applied in two different types of problems: regression as well as classification. While the regression approach tries to capture the behavior of the system, the classification tries to group and classify the system behavior in different subsystems.^[33]^

Principally, any kind of regression problem could be modeled as

(1) y=f(x)+ϵ,

where $f\left(x\right)$ represents a hidden function of input vector **x** and $\epsilon ∼N\left(0,\sigma_{n}^{2}\right)$ is an independent and identically distributed Gaussian noise function with zero mean and variance $\sigma_{n}^{2}$ originating from an observation y .

### 2.2 Linear Support Vector Regression

For a given training data set D of n observations, $D=\left\{\left(x_{i},y_{i}\right),i=1,2,...,n\right\}$ , where $x_{i}\in R^{d}$ represents a d‐dimensional input feature, $y_{i}$ represents a scalar target value, and n denotes the number of samples in the training set, Support Vector Regression (SVR) finds a d‐dimensional coefficient vector $w\in R^{d}$ and intercept coefficient b∈R such that the prediction given by $\left(w^{T}\varphi \left(x_{i}\right)+b\right)$ is close to target value $y_{i}$ . Here, the target value is the battery cycle life, and $x_{i}$ represents a vector of input features for battery sample i . The Linear SVR, subsequently, solves the following primal problem:^[34]^

(2) $$\min_{w,b}\left(\frac{1}{2}w^{T}w+C\sum_{i=1}max\left(0,\left|y_{i}-\left(w^{T}\varphi \left(x_{i}\right)+b\right)\right|-\epsilon \right)\right),$$

where the epsilon‐insensitive loss is used, which ignores errors smaller than ϵ , and C>0 is the regularization term. The dual problem is formulated as:^[35]^

(3) $$\min_{\alpha ,\alpha {}^{*}}\left(\frac{1}{2}{\left(\alpha -\alpha {}^{*}\right)}^{T}Q\left(\alpha -\alpha {}^{*}\right)+\epsilon e^{T}\left(\alpha +\alpha {}^{*}\right)-y^{T}\left(\alpha -\alpha {}^{*}\right)\right),$$

subject to $e^{T}\left(\alpha ,\alpha {}^{*}\right)=0,0<\alpha ,\alpha {}^{*}<C,i=1,...,n$ , where e is a vector of ones, $Q\in R^{n\times n}$ is a matrix with $Q_{ij}=\varphi {\left(x_{i}\right)}^{T}\varphi \left(x_{i}\right)$ . Finally, once the optimization problem is solved, the target value is predicted as:

(4) $$\sum_{i\in SV}\left(\alpha_{i}-\alpha_{i}{}^{*}\right)Q_{ij}+b\;,$$

where only support vectors (SV), i. e. samples that are within the margin, are considered.

### 2.3 Gaussian Process Regression

Gaussian Process Regression (GPR) is a non‐parametric machine learning methodology. Unlike other supervised machine learning algorithms that estimate the probability of parameters of a specific function, the GPR calculates all likely functions that are fitting to the observation data. This approach uses a Bayesian framework to do prediction by collecting prior knowledge and deriving a posterior probability hypothesis.

A GPR is typically defined by two key functions: the mean function $m\left(x\right)$ and the covariance function $k\left(x,x{}^{'}\right)$ which are defined as

(5) $$m\left(x\right)=E\left[f\left(x\right)\right],\;k\left(x,x^{'}\right)=E\left[\left(f\left(x\right)-m\left(x\right)\right)\left(f\left(x^{'}\right)-m\left(x^{'}\right)\right)\right].$$

By choosing the mean and covariance functions, one can write the Guassian process as:^[33]^

(6) $$f\left(x\right)∼GP(m\left(x\right),k\left(x,x^{'}\right)),$$

Furthermore, by summing the target value and noise distributions, one can simply include independently, identically distributed (i.i.d) Gaussian noise, $\epsilon ∼N\left(0,\sigma_{n}^{2}\right)$ , to the target value as:

(7) $$y∼GP(m\left(x\right),k\left(x,x^{'}\right)+\sigma_{n}^{2}I).$$

In supervised learning, locations with comparable observation values $x_{i}$ are predicted to have similar response (target) values *y_i_*. In GPR, this similarity is reflected by the covariance function, which determines how responses at one site $x_{i}$ are influenced by responses at other sites $x_{j}$ , $x_{i}\neq x_{j},i=1,2,...,n$ . Various kernel functions, with one or several hyper‐parameters, can be used to define the covariance function $k\left(x_{i},x_{j}\right)$ . Thus, the covariance function can be written as $k(x_{i},x_{j}|\theta )$ . For many conventional kernel functions, kernel variance *σ_f_* and characteristic length scale *σ_l_* are two common hyper‐parameters. The characteristic length scales describe how far the input values $x_{i}$ can be apart before the response values become uncorrelated. For any collection of input features $X=\left[x_{1},x_{2},...,x_{n}\right],$ the GPR defines a jointly Gaussian probability distribution $p(f\left(x_{1}\right),p(f\left(x_{2}\right),...,p\left(f\left(x_{n}\right)\right)$ . Therefore, from the GPR prior, the collection of training points and test points are joint multivariate Gaussian functions, with zero mean value, distributed as seen in Eq. 8,

(8) $$\left[\begin{array}{c} y\\ f{}^{*} \end{array}\right]∼N\left(0,\left[\begin{array}{cc} K\left(X,X\right)+\sigma_{n}^{2}I & K(X,X{}^{*})\\ K(X{}^{*},X) & K(X{}^{*},X{}^{*}) \end{array}\right]\right).$$

Given the number of training samples as n and number of test samples as $n{}^{*}$ , $K\left(X,X{}^{*}\right)$ denotes the $n\times n{}^{*}$ matrix of the computed covariances including all pairs of training and test points, and similarly for the other entries $K\left(X,X\right),K\left(X{}^{*},X\right),$ and $K\left(X{}^{*},X{}^{*}\right)$ . To improve the GPR's performance, the hyper‐parameters of the covariance function must be tuned. This can be achieved by maximizing the log marginal likelihood defined as:

(9) $$\log\left(y|X,\theta \right)=-\frac{1}{2}y^{T}{\left(K+\sigma_{n}^{2}I\right)}^{-1}y-\frac{1}{2}log\left|\begin{array}{c} K+\sigma_{n}^{2}I \end{array}\right|-\frac{n}{2}\log2\pi ,$$

where $-\frac{1}{2}y^{T}{\left(K+\sigma_{n}^{2}I\right)}^{-1}y$ is the data‐fit term, $-\frac{1}{2}log\begin{array}{c} K+\sigma_{n}^{2}I \end{array}$ is the complexity penalty term, and the $-\frac{n}{2}\log2\pi$ is the normalizing constant term. One can obtain the posterior distribution by limiting the joint prior distribution to the functions that are fitting to observed data points. Subsequently, predictions at test points could be made by computing the conditional distribution as (see e. g.^[33]^):

(10) $$p\left(f{}^{*}|X,y,X{}^{*}\right)∼N\left(\bar{f{}^{*}},cov\left(f{}^{*}\right)\right),$$

where

(11) $$\bar{f{}^{*}}=K\left(X{}^{*},X\right){\left[K\left(X,X\right)+\sigma_{n}^{2}I\right]}^{-1}y,$$

(12) $$cov\left(f{}^{*}\right)=K\left(X{}^{*},X{}^{*}\right)-K\left(X{}^{*},X\right){\left[K\left(X,X\right)+\sigma_{n}^{2}I\right]}^{-1}K\left(X,X{}^{*}\right).$$

## 3 Methodologies

**Figure 1:**
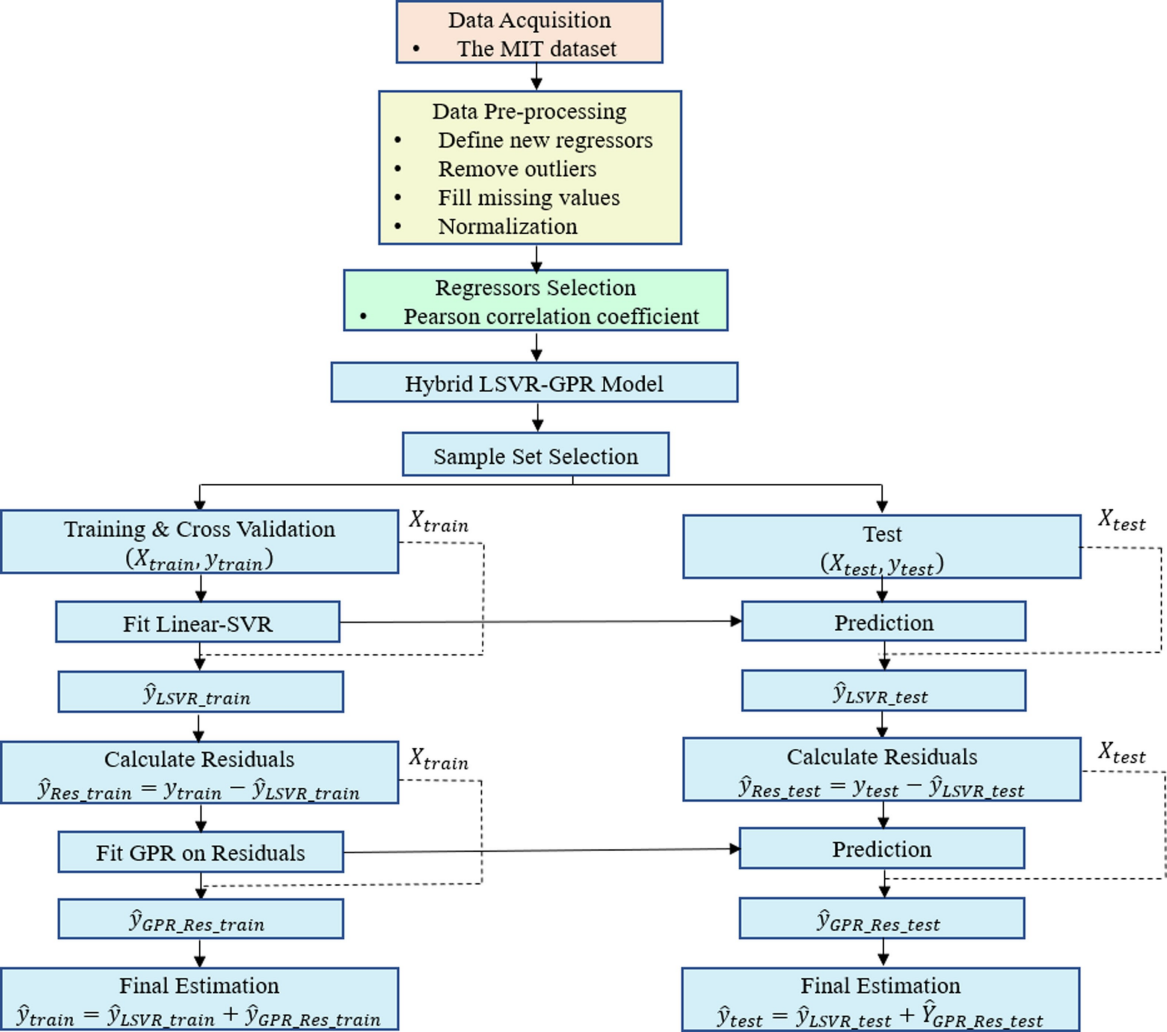
Cycle life estimation procedure.

The major purpose of this study is to predict Li‐ion battery cycle life at an early stage of battery usage. More specifically, we hypothesize that merging the LSVR and GPR models could yield better results than state‐of‐the‐art methodology,^[32]^ while still using the same data. Figure 1 depicts the procedure and steps for estimating cycle life, which include data description, data pre‐possessing, feature selection, and model development, all of which are covered in detail in the following subsections.

### 3.1 Data Description

**Figure 2:**
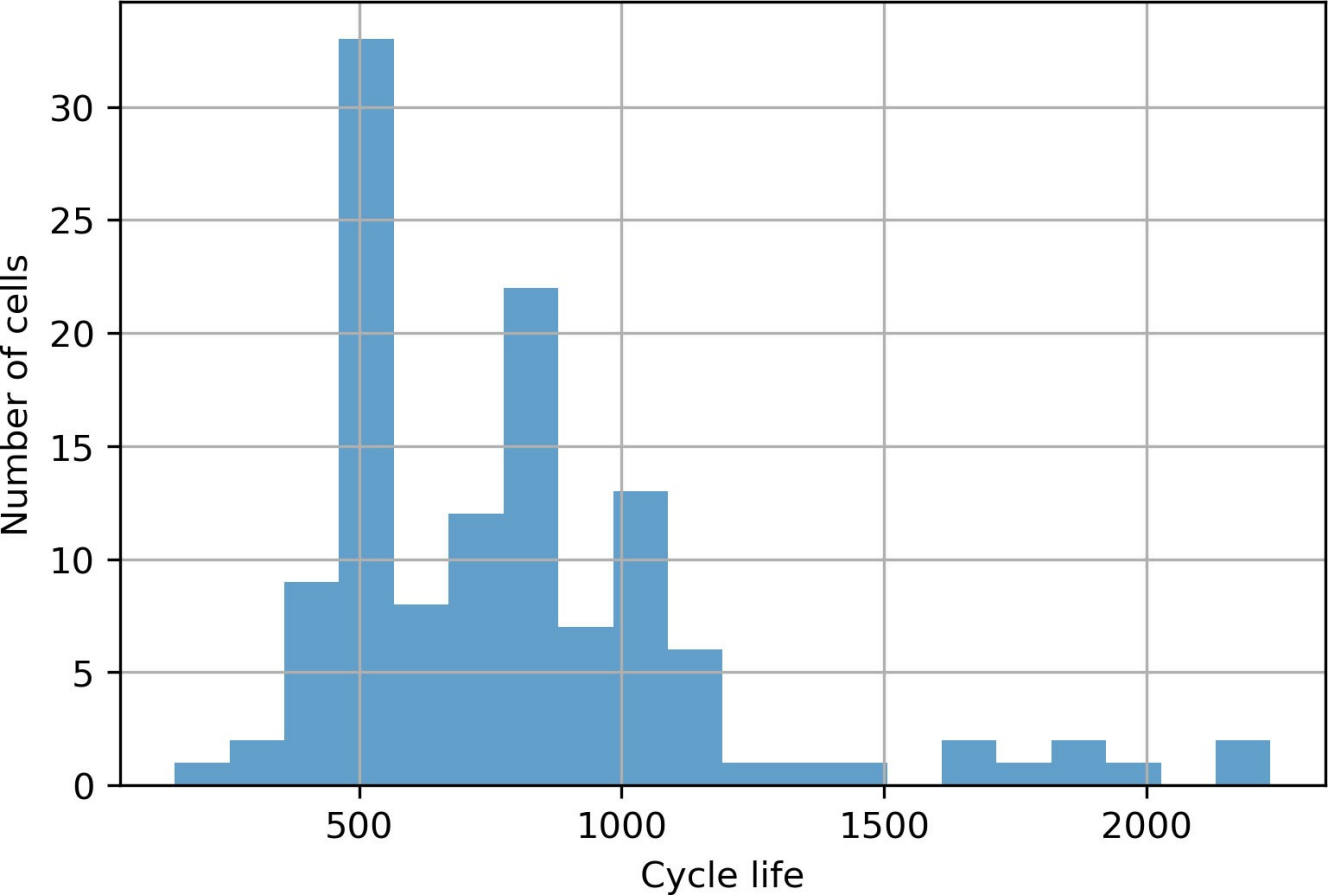
Cycle‐life histogram for 124 battery samples in the MIT dataset.

Reis et al.^[36]^ reviewed over 30 datasets associated with Li‐ion batteries. The MIT data set^[32]^ consisting of cycling data for 124 LFP/ graphite cells (A 123 systems, model APR18650M1A, 1.1 Ah nominal capacity) was used in this work. All cells were charged using a variety of multi‐step fast charging methodologies, then discharged at a constant current. For all cycles, the ambient temperature was fixed to 30 °C. Continuous data including voltage, current, battery temperature, and internal resistance were collected as the battery cells were cycled to end of life (EOL), defined as 80 % of their initial capacity. The cycle‐life histogram for 124 cell samples ranging from 150 to 2300 cycles is shown in Figure 2.

### 3.2 Data Pre‐Processing

In ML applications, data pre‐processing is critical for improving data quality and prediction accuracy. Generally, it includes removing outliers, filling missing values, time‐domain synchronization, and normalization.^[37]^ In this context, some battery samples from noisy channels as well as some batteries that did not reach 80 % capacity were removed. Two samples with outliers were noticed in the capacity fade curve for the first 100 cycles. The detected outliers were removed, and the missing data are then filled up using interpolated values. Finally, the whole data set was normalized using the z‐score normalization method^[38]^ as:

(13) $$Z=\frac{x-\mu }{\sigma },$$

where Z is the standard score, x is the observed value, μ is the sample mean, and σ is the sample standard deviation.

### 3.3 Feature Selection

Normally, machine learning applications contain plenty of input features in the dataset. While some of these features might have good predictive strength, the presence of non‐informative features can add uncertainty to the predictions. Therefore, when it comes to creating a machine learning model, feature selection is crucial to minimize the number of input variables, to lower the computational cost of modeling, and to increase the model's performance. The two fundamental types of feature selection approaches are supervised and unsupervised procedures. The distinction is whether or not the features are chosen based on the target variable. Unsupervised feature selection strategies, such as those that remove redundant variables using correlation, disregard the target variable. Approaches that use the target variable, such as methods that eliminate irrelevant variables, are supervised feature‐selection techniques. In this section, an unsupervised method was used to remove redundant features. Features with high correlation have approximately the same influence on the observed output. Therefore, when two features have a high correlation, one of them might be dropped without losing relevant information for predicting the output of interest.

Before eliminating redundant features, additional features were added to the available ones developed by Severson et al.^[32]^ All features with their respective definition are listed in Table 1. Below is a description of how the features are derived:^[32]^

(14) $$\Delta Q\left(V\right)=Q_{100}\left(V\right)-Q_{10}\left(V\right),\Delta Q\left(V\right)\in R^{p},\;\Delta T\left(V\right)=T_{100}\left(V\right)-T_{10}\left(V\right),\Delta T\left(V\right)\in R^{p},$$

(15) $$\bar{\Delta Q}\left(V\right)=\frac{1}{p}\sum_{i=1}^{p}\Delta Q\left(V\right),$$

(16) $$b{}^{*}=\underset{b}{\arg\;\min}\frac{1}{m}{∥q-Nb∥}_{2}^{2},$$

**Table 1:** List of input features.

	Features description	Symbol	Equation
▵*Q*100 10(*V*) features	Minimum	x_min	$\log\left(\left|min\left(\Delta Q\left(V\right)\right)\right|\right)$
		
	Mean	x_mean	$\log\left(\left|\bar{\Delta Q}\left(V\right)\right|\right)$
		
	Variance	x_var	$\log\left(\left|\frac{1}{p-1}\sum_{i=1}^{p}{\left(\Delta Q\left(V\right)-\bar{\Delta Q}\left(V\right)\right)}^{2}\right|\right)$
		
	Skewness	x_skew	$\log\left[\left|\frac{\frac{1}{p}\sum_{i=1}^{p}{\left(\Delta Q\left(V\right)-\bar{\Delta Q}\left(V\right)\right)}^{3}}{{\left(\sqrt{\sum_{i=1}^{p}{\left(\Delta Q\left(V\right)-\bar{\Delta Q}\left(V\right)\right)}^{2}}\right)}^{3}}\right|\right]$
		
	Kurtosis	x_kurt	$\log\left[\left|\frac{\frac{1}{p}\sum_{i=1}^{p}{\left(\Delta Q\left(V\right)-\bar{\Delta Q}\left(V\right)\right)}^{4}}{{\left(\sqrt{\sum_{i=1}^{p}{\left(\Delta Q\left(V\right)-\bar{\Delta Q}\left(V\right)\right)}^{2}}\right)}^{2}}\right|\right]$
			
Discharge capacity fade curve features	Slope of the linear fit to the capacity fade curve, cycles 2 to 100	x_slopeDC	the first value in the vector **b** ^✶^ where *d*=99
	Intercept of the linear fit to capacity fade curve, cycles 2 to 100	x_constDC	the second value in the vector **b** ^✶^ where *d*=99
	Slope of the linear fit to the capacity fade curve, cycles 91 to 100	x_slope90	the first value in the vector **b** ^✶^ where *d*=10
	Intercept of the linear fit to capacity fade curve, cycles 91 to 100	x_const90	the second value in the vector **b** ^✶^ where *d*=10
	Discharge capacity, cycle 2	x_QD2	*Q*(*n*=2)
	Difference between max discharge capacity and cycle 2	x_Qdiff	max_*n*_ *Q*(*n*) − *Q*(*n*=2)
	Discharge capacity, cycle 100	x_QD100	*Q*(*n*=100)
			
Other features	Average charge time, first 5 cycles	x_chargetime	$\frac{1}{5}\sum_{i=1}^{6}$ Charge Time_*i*_
	Maximum temperature, cycles 2 to 100	x_maxT	max_*n*_ *T* (*n*)
	Minimum temperature, cycles 2 to 100	x_minT	min_*n*_ *T* (*n*)
	Integral of temperature over time, cycles 2 to 100	x_tempint	$\int_{t_{2}}^{t_{100}}T\left(t\right)dt$
	Internal resistance, cycle 2	x_IR2	IR (*n*=2)
	Minimum internal resistance, cycles 2 to 100	x_IRmin	min_*n*_IR(*n*)
	Internal resistance, difference between cycle 100 and 2	x_IRdiff	IR (*n*=100)−IR(*n*=2)
			
Features added by this work	Variance of ▵*T* (*V*)	x_varT	$\log\left(\left|\frac{1}{p-1}\sum_{i=1}^{p}{\left(\Delta T\left(V\right)-\bar{\Delta T}\left(V\right)\right)}^{2}\right|\right)$
		
	Mean of dVdQ curve at cycle 100	x_mean dVdQ	$\log\left(\left|{\bar{dVdQ}}_{100}\right|\right)$
	Mean of dVdT curve at cycle 100	x_mean dVdT	$\log\left(\left|{\bar{dQdT}}_{100}\right|\right)$
	Mean of dQdV curve at cycle 100	x_mean dQdV	$\log\left(\left|{\bar{dQdV}}_{100}\right|\right)$
	Coulombic efficiency at cycle 2	x_CE2	$\frac{QD}{QC}\left(n=2\right)$
	Coulombic efficiency at cycle 100	x_CE100	$\frac{QD}{QC}\left(n=100\right)$
	Variance of Coulombic efficiency cycle 2 to 100	x_CEvar	$\log\left(\left|\frac{1}{p-1}\sum_{i=1}^{p}{\left(CE_{2-100}-\bar{CE_{2-100}}\right)}^{2}\right|\right)$

where m is the number of cycles in the prediction, $q\in R^{m}$ is a vector of discharge capacities as a function of the cycle number, $N\in R^{m\times 2}$ is a matrix with the first column containing cycle numbers and the second column containing a vector of ones, and $b\in R^{2}$ is a coefficient vector.

**Figure 3:**
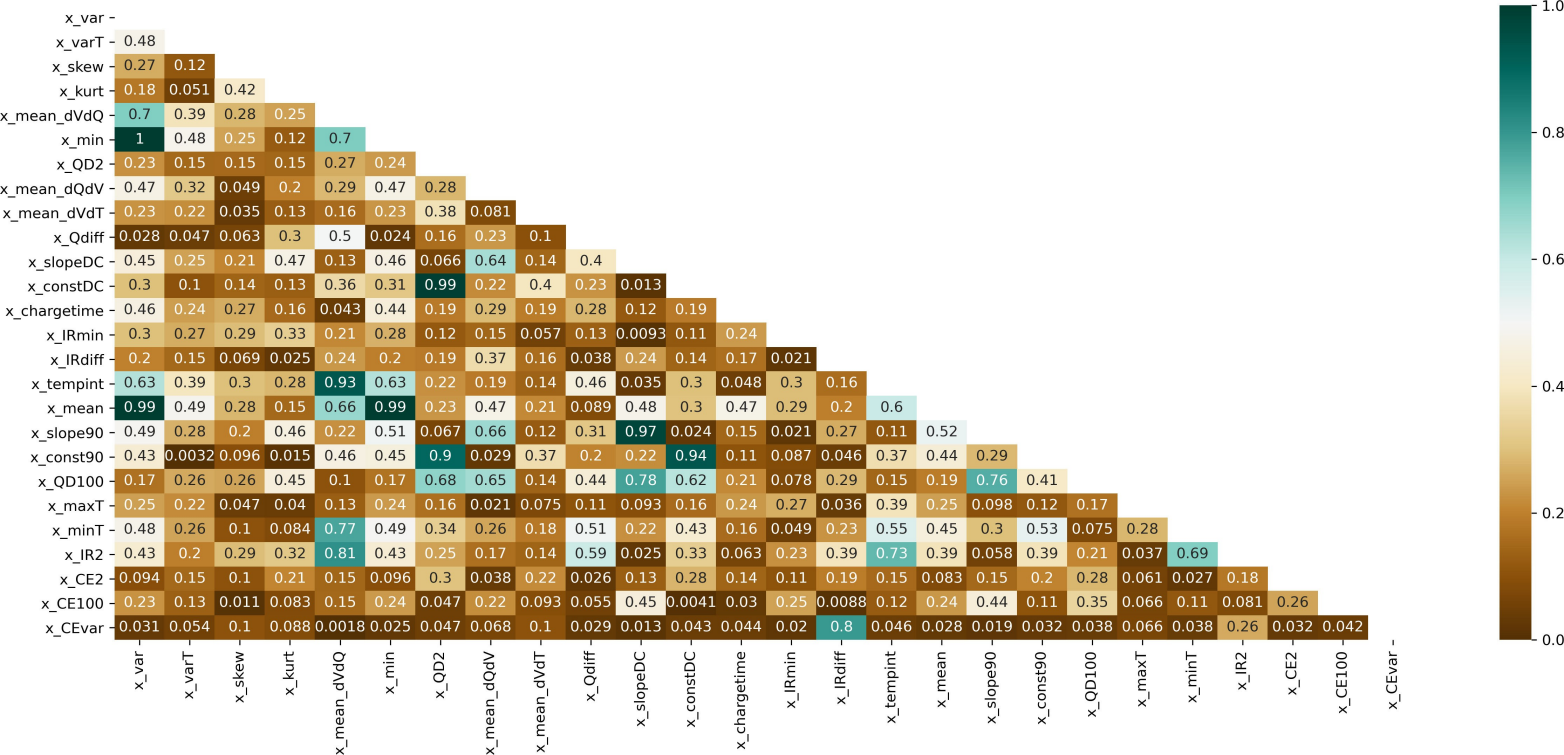
Triangle Correlation Heatmap for the dataset.

Figure 3 shows the correlation heat‐map including all features. To remove redundant input variables, columns with correlation greater than 0.9 were dropped. As a result, six features of twenty‐six were removed.

### 3.4 Model Development

In this section, a comprehensive data‐driven model was employed to predict battery cycle life before more severe capacity degradation phenomenon occurs. To this end, two hybrid models combining a LSVR and a GPR model were developed. While the LSVR model was used to forecast battery cycle life, the GPR model was used to model the cycle life residual, which is defined as the difference between the real cycle life and the LSVR model's predicted cycle life. Severson et al.^[32]^ utilized a linear model, and used the lasso and elastic net techniques for regularization to avoid over‐fitting. They used four‐fold cross‐validation and Monte Carlo sampling for tuning hyper‐parameters. Because recreating the same results would be difficult, the LSVR model, which employs the linear kernel, is used in this study. The GPR model was tested in the form of two different models: model A and model B. As illustrated in Figure 1, the final predictions were obtained by adding the LSVR model's predicted cycle life and the GPR model's predicted cycle life residual. The final models are therefore called hybrid model A and hybrid model B. It is worth noting that this design is theoretically equal to setting the LSVR model as a mean function of the GPR model.

**Figure 4:**
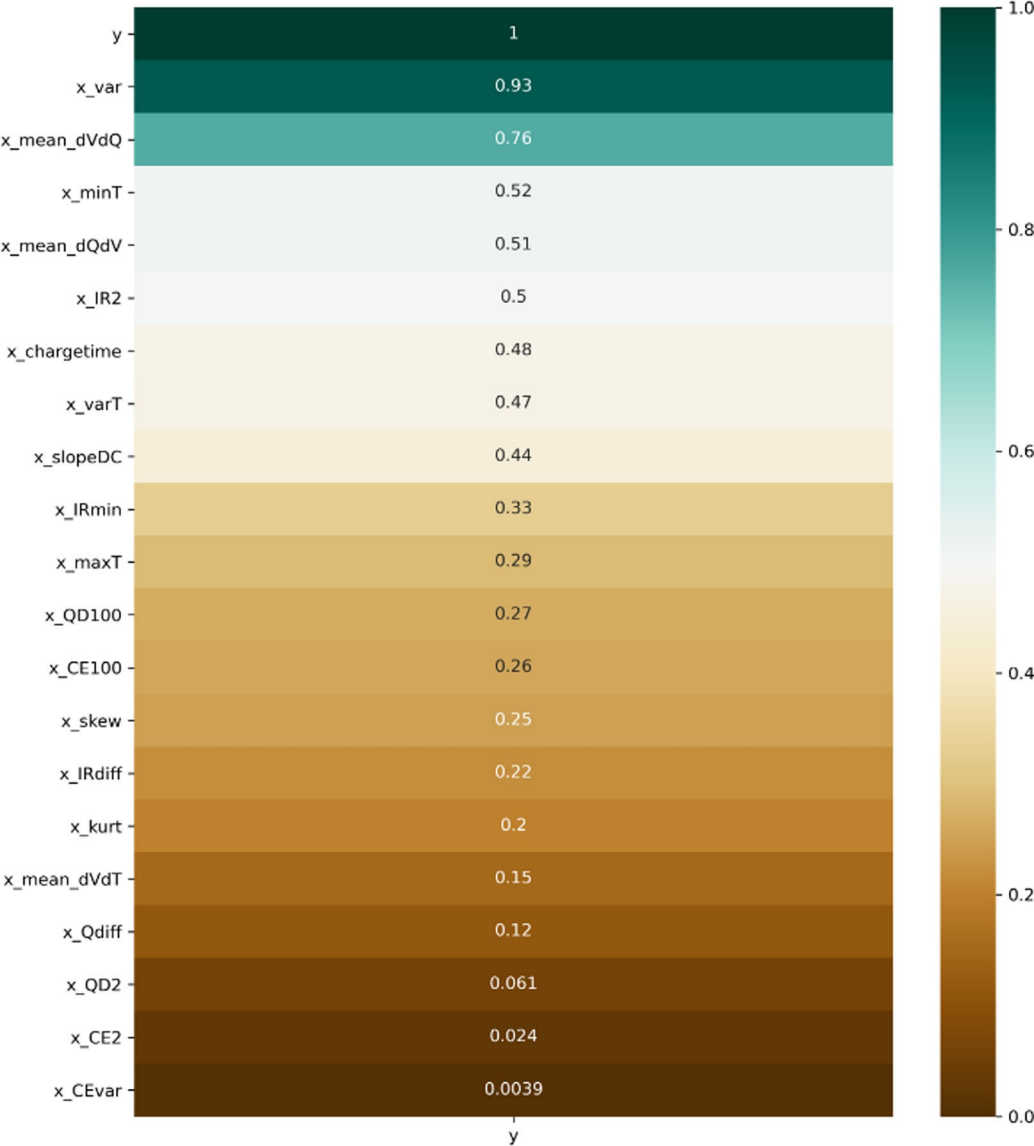
Pearson correlation coefficients between individual regressors and battery cycle lifes.

In section [FS], an unsupervised feature selection strategy was used to remove redundant features. In this section, the filter feature selection method was used to select the most relevant features. The filter‐based feature selection method is a supervised method which uses statistical techniques to asses the relevance of features and target variable outside of the predictive models.^[39]^ The absolute valued Pearson correlation coefficient, as the most commonly used ranking criterion in the filter methods, was employed to select the most relevant features correlated to the target values. It determines the linear relationship between the feature x and the target y , as:

(17) $$r_{xy}=\frac{\sum_{i=1}^{n}\left(x_{i}-\bar{x}\right)\left(y_{i}-\bar{y}\right)}{\sqrt{\sum_{i=1}^{n}{\left(x_{i}-\bar{x}\right)}^{2}\sqrt{\sum_{i=1}^{n}{\left(y_{i}-\bar{y}\right)}^{2}}}},$$

where $x_{i}$ and $y_{i}$ denote the i‐th sample of feature x and the target y , and $\bar{x}$ and $\bar{y}$ are the independent and dependent sample means, respectively. Figure 4 shows the listed computed Pearson coefficients between the remaining features and the cycle life value. A threshold of 0.5 was utilized to filter the relevant features to be used as an input variables in the LSVR model, leading to the final choice of x_var , x_mean_dVdQ , x_minT , x_mean_dQdV , as well as x_IR2 .

**Figure 5:**
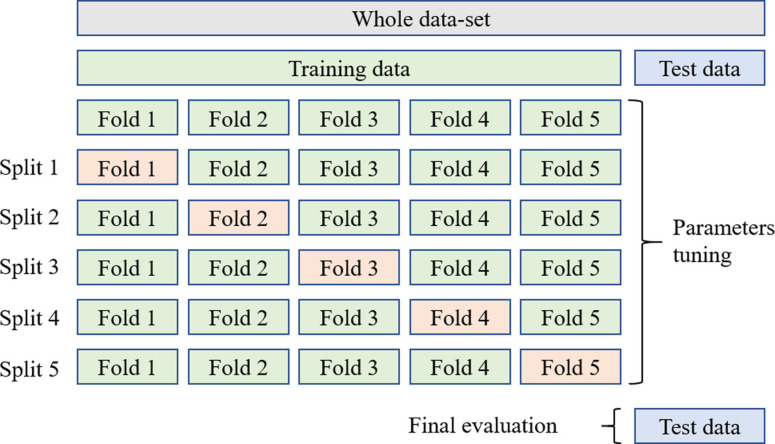
5‐fold cross validation procedure.

Learning the parameters of a prediction function and testing it on the same data set is a fundamental error that can result in over‐fitting. In machine learning applications, the common practice is to divide the entire data into three sets of data, i. e. training, cross‐validation and testing, 60 : 20 : 20. It is well‐known that the basic idea of cross‐validation is to split the training set into two disjoint sets, one which is actually used for training, and the other, the validation set, which is used to monitor the performance of the trained model. The answer to the question on what the optimal number of the chosen folds would be, is more based on experimental rather than theoretical studies. One approach would be to choose the so‐called leave‐one‐out cross‐validation (LOO‐CV), i. e. an extreme case of k‐fold cross‐validation obtained for k=n, the number of training cases. While his approach can be computational heavy, but the typical values for k are often in the range 3 to 10. In this work, the 80/20 training/test split on the data‐set was used. Furthermore, the training set was split in to 5 smaller sub‐sets, meaning that the 5‐fold cross‐validation was performed. Figure 5 depicts the procedure for k ‐fold cross‐validation, in which a model is trained using k ‐1 of folds as training data and the resulting model is validated on the remaining data. After fitting the model using the training data and thereafter cross‐validating it, the model was evaluated using the test set. We evaluated various cross‐validation with different k‐folds (k=1,2,...5 ), with the results showing that our choice of 5‐fold cross‐validation had the lowest error.

### 3.5 Model A

**Figure 6:**
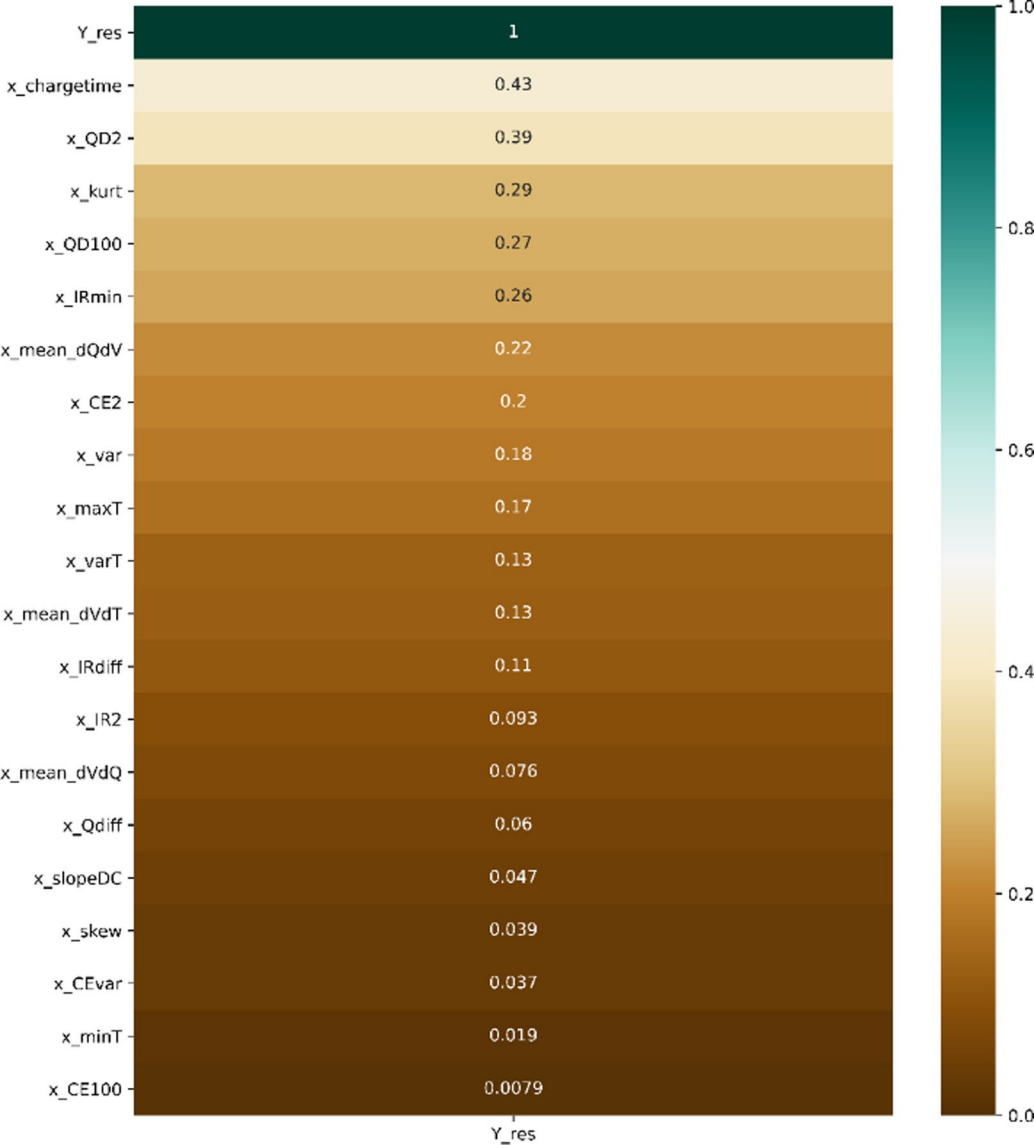
Pearson correlation coefficients between individual regressors and battery cycle life residual.

It is worth noting that the covariance function must be carefully chosen or built since it determines the GPR's functionality. As discussed earlier, the covariance function determines how responses at one site $x_{i}$ are influenced by responses at other sites $x_{j}$ , $x_{i}\neq x_{j},i=1,2,...,n$ . In model A, firstly, relevant features with the cycle life residual were filtered using the Pearson correlation coefficient. The Pearson coefficients vary from 0.0079 to 0.43, as shown in Figure 6. As a result, a 0.25 threshold was set to filter the relevant features, and five features were chosen to be used in Model A. Then, five different isotropic kernel functions, i. e. with the same length scale hyper‐parameter, see section [GPR], for each feature, were used in the GPR model. The isotropic squared exponential (radial basis function‐ RBF) kernel function is one of the most common used covariance functions, and defined as:

(18) $$k_{SE}\left(x_{i},x_{j}|\theta \right)=\sigma_{f}^{2}\exp\left(-\frac{{\left(x_{i}-x_{j}\right)}^{T}\left(x_{i}-x_{j}\right)}{2\sigma_{l}^{2}}\right),\;$$

where $\sigma_{l}$ is the characteristic length scale, and $\sigma_{f}$ is the signal standard deviation. The isotropic Matern 3/2 kernel is defined by:

(19) $$k_{Matern32}\left(x_{i},x_{j}|\theta \right)=\sigma_{f}^{2}\left(1+\frac{\sqrt{3}}{\sigma_{l}}r\right)\exp\left(-\frac{\sqrt{3}}{\sigma_{l}}r\right),$$

where $r=\sqrt{{\left(x_{i}-x_{j}\right)}^{T}\left(x_{i}-x_{j}\right)}$ . Similarly, the Matern 5/2, rational quadratic (RatQuad), exponential (Exp), and linear kernel functions are defined as following equations:

(20) $$k_{Matern52}\left(x_{i},x_{j}|\theta \right)=\sigma_{f}^{2}\left(1+\frac{\sqrt{5}}{\sigma_{l}}r+\frac{5r^{2}}{3\sigma_{l}^{2}}\right)\exp\left(-\frac{\sqrt{5}}{\sigma_{l}}r\right),$$

(21) $$k_{RatQuad}\left(x_{i},x_{j}|\theta \right)=\sigma_{f}^{2}\left(1+\frac{r^{2}}{2\alpha \sigma_{l}^{2}}\right),$$

(22) $$k_{Exp}\left(x_{i},x_{j}|\theta \right)=\sigma_{f}^{2}\left(\frac{r}{\sigma_{l}}\right).$$

### 3.6 Model B

In model B, in contrast to model A, the entire input features were used where kernels with different length scales $\sigma_{l}$ were used for each feature. Here, $x_{il}$ denotes a single feature l of sample i and $x_{jl}$ denotes a single feature l of sample j where $x_{i}\neq x_{j}$ , i=1,2,...,n , and l=1,2,...,d . The automatic relevance determination (ARD) structure was implemented to extract the highly relevant input features for cycle life estimation. In principle, through using model B, irrelevant features might be limited by setting large length scales for them, resulting in a reduced and descriptive dataset.^[40]^

Five alternative ARD‐kernels were investigated to assess the GPR performance with model B, just as they were with model A. The ARD Squared Exponential kernel is defined as:

(23) $$k_{SE\_ARD}\left(x_{i},x_{j}|\theta \right)=\sigma_{f}^{2}\exp\left(-\frac{1}{2}\sum_{l=1}^{d}\frac{{\left(x_{il}-x_{jl}\right)}^{2}}{\sigma_{l}^{2}}\right).$$

The ARD Matern 3/2 is defined as:

(24) $$k_{Matern32\_ARD}\left(x_{i},x_{j}|\theta \right)=\sigma_{f}^{2}\left(1+\sqrt{3}r\right)\exp\left(-\sqrt{3}r\right),$$

where $r=\sqrt{\sum_{l=1}^{d}\frac{{\left(x_{il}-x_{jl}\right)}^{2}}{\sigma_{l}^{2}}}$ . Similarly, the ARD Matern 5/2, ARD rational quadratic (RatQuad), ARD exponential (Exp), and ARD linear kernel functions are defined in the following equations:

(25) $$k_{Matern52\_ARD}\left(x_{i},x_{j}|\theta \right)=\sigma_{f}^{2}\left(1+\sqrt{5}r+\frac{5r^{2}}{3}\right)\exp\left(-\sqrt{5}r\right),$$

(26) $$k_{RatQuad\_ARD}\left(x_{i},x_{j}|\theta \right)=\sigma_{f}^{2}{\left(1+\frac{1}{2\alpha }\sum_{l=1}^{d}\frac{{\left(x_{il}-x_{jl}\right)}^{2}}{\sigma_{l}^{2}}\right)}^{-\alpha },$$

(27) $$k_{Exp}\left(x_{i},x_{j}|\theta \right)=\sigma_{f}^{2}\left(-r\right).$$

### 3.7 Model Assessment

To evaluate the outcomes, the predicted cycle lifes should be compared to the actual cycle lifes from the observation data. In this sense, this work employs two distinct metrics, root mean square error (RMSE) and mean percent error (% err), similar to those employed by Severson et al.^[32]^ The metrics are defined as:

(28) $$RMSE=\sqrt{\frac{1}{n}\sum_{i=1}^{n}{\left(y_{i}-{\hat{y}}_{i}\right)}^{2}},$$

(29) $$\%err=\frac{1}{n}\sum_{i=1}^{n}\frac{\left|y_{i}-{\hat{y}}_{i}\right|}{y_{i}}\times 100.$$

## 4 Results and Discussion

Section 3.4 covered the design of the developed hybrid data‐driven models. The major point of interest in this study has been to improve the accuracy of the predicted remaining useful life for the studied batteries. Different statistical and data‐driven‐models were examined as described in chapter 3. The GPR model was used to forecast the cycle life residuals after subtracting the predicted cycle life from the observed cycle life values using the LSVR model. The hybrid models were developed in two forms: hybrid model A and hybrid model B. The key differences between them are the method of input feature selection and the type of kernels used in the covariance matrix for each case.

**Figure 7:**
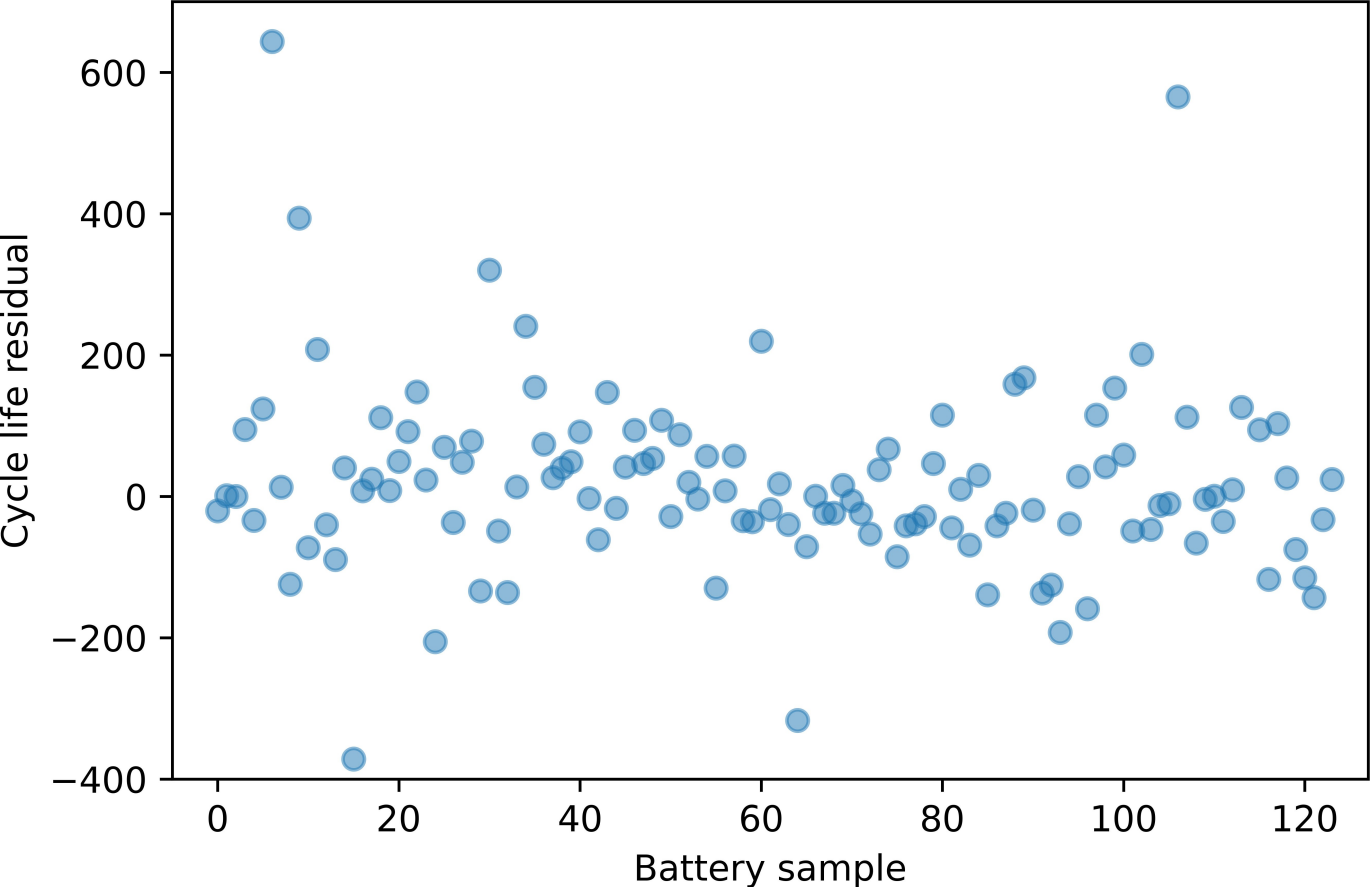
Distribution of cycle life residual data for all the battery samples.

Figure 7 shows the cycle life residual data distribution across all battery samples. The goal here is to use the GPR model to estimate the cycle life residual for each of the samples. To this end, a GPR model with alternative kernel functions was examined, as described in section 3.4. Although the squared exponential (SE) kernel function is powerful for machine learning applications, one drawback could be the smoothness of the predicted model which can exclude specific behaviors in the studied data. Here, the Matern class of covariance with or without ARD (Automatic Relevance Determination) can be of use. This class of kernel functions use Bessel functions and additional positive hyperparameters. The scaling parameter is chosen so that for an infinitely large scale factor, the kernel will converge to the ordinary SE covariance function. Thus, there is a trade‐off between the smoothness and required hardness when choosing the right value for the scaling parameter. Low values (e. g. $\frac{1}{2}$ ) would be too rough, whereas high values (e. g. $\frac{7}{2}$ ) would be too smooth. The results provided in Table 2 clearly indicate this fact.

**Table 2:** Performance of model A using five different isotropic kernels.

Hybrid model A	RMSE		Mean Percent Error (%)	
	Training	Test	Training	Test
RBF	12.8	180.8	1.1	9.3
Matern 32	13.2	177.2	1.1	8.6
Matern 52	13.0	178.5	1.1	8.9
RatQuad	13.5	179.2	1.1	8.6
Exponential	13.8	177	1.1	8.3

Table 2 lists the prediction accuracy of hybrid model A using the RBF, Matern 3/2, Matern 5/2, rational quadratic, and exponential kernels. Despite the fact that the exponential kernel had the highest RMSE for the training set among all the kernels, it was chosen to represent model A since it had the lowest RMSE and %err for the test set. The hybrid model A has the advantage of keeping the %err for both the training and test sets below 10 %, despite the kernel function used in the GPR model.

Similarly, Table 3 lists the performance of hybrid model B with five different ARD kernels. Using all ARD kernels in the GPR model, hybrid model B, like hybrid model A, is capable of keeping the %err below 10 %. Among these, the model using the exponential kernel has the best performance, with RMSE of 16.6 and 152, and %err of 1.4 and 8.2 for the training and test set, respectively.

**Table 3:** Performance of model B using five different ARD‐kernels.

Hybrid model B	RMSE		Mean Percent Error (%)	
	Training	Test	Training	Test
RBF_ARD	21.4	173.2	2.0	9.2
Matern 32_ARD	19.2	176.5	1.8	9.7
Matern 52_ARD	20.2	176.9	1.9	9.7
RatQuad_ARD	20.2	175.8	1.9	9.6
Exponential_ARD	16.6	152	1.4	8.2

**Figure 8:**
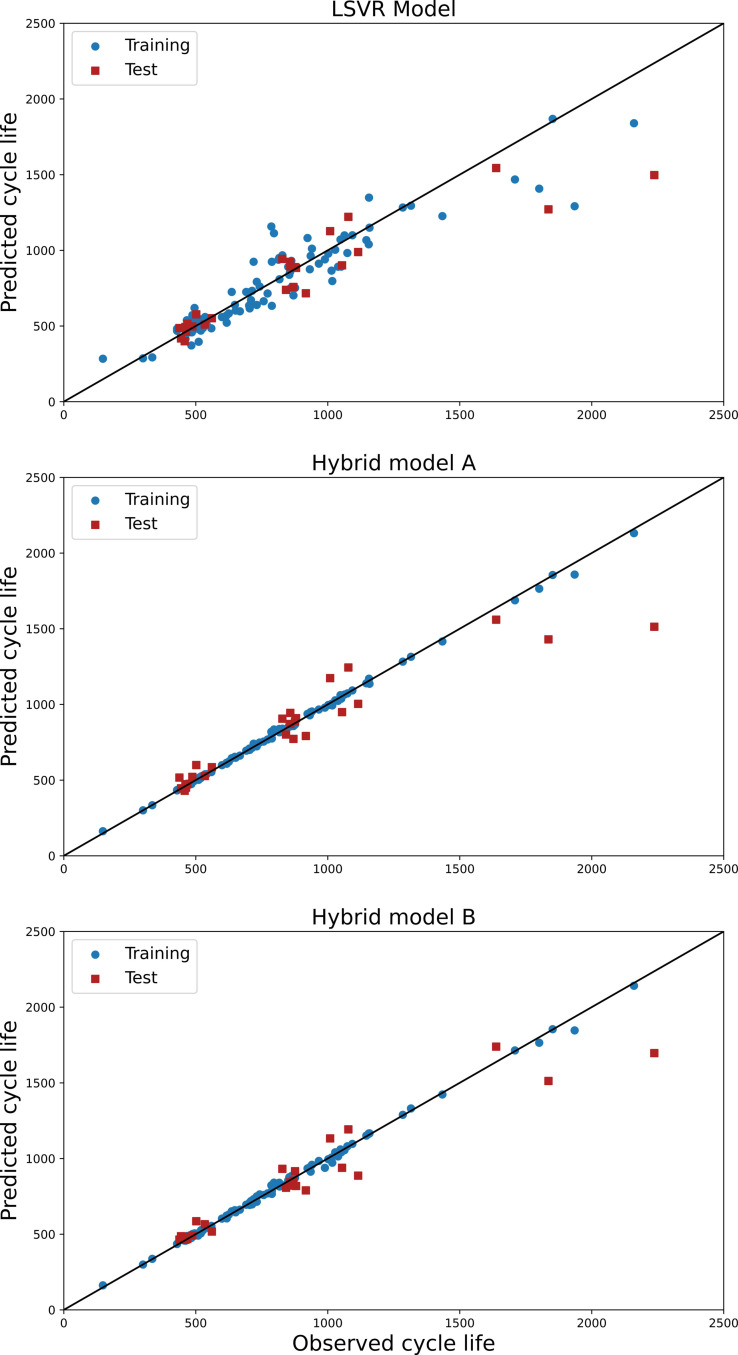
Predicted cycle life versus the real cycle life for the LSVR model, the hybrid model A, and the hybrid model B.

The final form of the hybrid models A and B is accepted as those with the exponential kernel in the GPR model. The predicted versus real cycle lifes for the LSVR, hybrid model A, and hybrid model B are depicted in Figure 8, with the blue points representing training samples and the red points representing test points. The more linear the distribution is, the higher the prediction performance. The hybrid models are clearly more linearly distributed, implying that the predicted cycle lives are closer to the real values.

Performance prediction of the LSVR model, hybrid model A, and hybrid model B was thereafter evaluated. The models were tested using five different kernels, and the best results were chosen and compared with Severson et al.^[32]^ Two metrics, the RMSE and %err, were used to evaluate the prediction performance of the models.

Table 4 benchmarks the current work with the linear model developed by Severson et al.^[32]^ who developed three separate models: the “Variance”, the “Discharge”, and the “Full” model, based on the feature types selected from different subgroups, and predicted and classified cells by cycle life. They reported their results in two ways (including and excluding an outlier sample that reached the end of life before cycle 100) for two sets of test: test 1 and test 2. They obtained high error values for the entire training, test 1, and test 2 sets using the “Variance” model, with RMSE values greater than 100 and %err values greater than 10. They had slightly better results using the “Discharge” model with %err of 8.6 for test 2 and RMSE values less tha 100 for training and test 1. Using their “Full” model, they got 118 for RMSE and 14.1 for %err for test 1 including the outlier. They achieved high prediction accuracy after excluding that sample, with an RMSE of 100 and a %err of 7.5 for test1. However for test 2, even excluding the outlier, the “Full” model failed to keep %err below 10.

**Table 4:** Benchmarking the models.

Benchmark		RMSE			Mean Percent Error (%)		
	Model	Training	Test 1	Test 2	Training	Test 1	Test 2
Severson	Variance	103	138 (138)	196	14.1	14.7 (13.2)	11.4
et al.^[32]^	Discharge	76	91 (86)	173	9.8	13 (10.1)	8.6
	Full	51	118 (100)	214	5.6	14.1 (7.5)	10.7

	Model	Training	Test		Training	Test	
This work	LSVR	136.4	191.2	–	12.2	12.6	–
(excludingadded features)	Hybrid model A	15.9	167.9	–	1.3	10.2	–
	Hybrid model B	17.7	167.6	–	1.4	10.2	–

	Model	Training	Test		Training	Test	
							
This work	LSVR	128.4	204.8	–	10.9	10.6	–
(including added features)	Hybrid model A	13.8	177.0	–	1.1	8.3	–
	Hybrid model B	16.6	152.0	–	1.4	8.2	–

In this study, results were reported, as shown in Table 4, both with and without the added input features. Without the added input features, the LSVR model shows comparable % err values both for training (12.2 %) and test (12.6 %) set. However, when comparing the LSVR model to the hybrid models A and B, the latter perform better, especially on training data. With the new input features added to this study, hybrid model A outperforms all other models in terms of the RMSE (13.8) and %err (1.1 %) for the training set, while hybrid model B, with the RMSE and %err of 152 and 8.2, showed the best performance for the test data. Both models offer two key advantages over the other models: the first is that they keep the %err below 10 % for both the training and test sets, and the second is that the metrics of the training and test sets are not drastically different.

All of the computations were done on a personal computer (Intel(R) Core(TM) i9‐10885H CPU @ 2.40 GHz). It's worth mentioning that loading the data takes the longest time. The LSVR model takes 0.29 seconds, while the hybrid models A and B with exponential kernels take 8 and 11 seconds to run, respectively.

## 5 Conclusion and Future Work

Battery lifetime prediction at an early stage of cycling is critical for safe operation, considering the rapid technology development, and need for accurate state of health (SOH) monitoring in EV applications. Most data‐driven models described in literature need data relating to at least 25 % of the aging process in order to properly predict battery lifetime. In this paper, a hybrid data‐driven model combining the LSVR and GPR is proposed to effectively predict battery cycle life using data from only the first 100 cycles.

Although the presented approach has shown the inherent potential of using data‐driven approaches for describing and predicting the complex physical processes such as estimation of the Li‐ion battery cycle life, the data greediness of these methods still calls for need of further research in the field. A smart combination of a physical reduced order model (ROM) with less parameters to be identified together with real as well as synthetic data would be one option track for future work.

## Conflict of interest

The authors declare no conflict of interest.

6

## Data Availability

The data that support the findings of this study are available from the corresponding author upon reasonable request.
